# Nrf2 Is a Key Regulator on Puerarin Preventing Cardiac Fibrosis and Upregulating Metabolic Enzymes UGT1A1 in Rats

**DOI:** 10.3389/fphar.2018.00540

**Published:** 2018-06-06

**Authors:** Shao-Ai Cai, Ning Hou, Gan-Jian Zhao, Xia-Wen Liu, Ying-Yan He, Hai-Lin Liu, Yong-Quan Hua, Li-Rong Li, Yin Huang, Cai-Wen Ou, Cheng-Feng Luo, Min-Sheng Chen

**Affiliations:** ^1^Department of Cardiology, Guangdong Provincial Biomedical Engineering Technology Research Center for Cardiovascular Disease, Sino-Japanese Cooperation Platform for Translational Research in Heart Failure, Zhujiang Hospital, Southern Medical University, Guangzhou, China; ^2^The Second Affiliated Hospital of Guangzhou Medical University, Guangzhou, China; ^3^School of Pharmaceutical Sciences and The Fifth Affiliated Hospital, Guangzhou Medical University, Guangzhou, China; ^4^Guangzhou Institute of Cardiovascular Disease, The Second Affiliated Hospital of Guangzhou Medical University, Guangzhou, China

**Keywords:** puerarin, cardiac fibrosis, Nrf2, UGT1A1, metabolic feedback loop

## Abstract

Puerarin is an isoflavone isolated from *Radix puerariae*. Emerging evidence shown that puerarin possesses therapeutic benefits that aid in the prevention of cardiovascular diseases. In this study, we evaluated the effects of puerarin on oxidative stress and cardiac fibrosis induced by abdominal aortic banding (AB) and angiotensin II (AngII). We also investigated the mechanisms underlying this phenomenon. The results of histopathological analysis, as well as measurements of collagen expression and cardiac fibroblast proliferation indicated that puerarin administration significantly inhibited cardiac fibrosis induced by AB and AngII. These effects of puerarin may reflect activation of Nrf2/ROS pathway. This hypothesis is supported by observed decreases of reactive oxygen species (ROS), decreases Keap 1, increases Nrf2 expression and nuclear translocation, and decreases of collagen expressions in cardiac fibroblasts treated with a combination of puerarin and AngII. Inhibition of Nrf2 with specific Nrf2 siRNA or Nrf2 inhibitor brusatol attenuated anti-fibrotic and anti-oxidant effects of puerarin. The metabolic effects of puerarin were mediated by Nrf2 through upregulation of UDP-glucuronosyltransferase (UGT) 1A1. The Nrf2 agonist tBHQ upregulated protein expression of UGT1A1 over time in cardiac fibroblasts. Treatment with Nrf2 siRNA or brusatol dramatically decreased UGT1A1 expression in puerarin-treated fibroblasts. The results of chromatin immunoprecipitation–qPCR further confirmed that puerarin significantly increased binding of Nrf2 to the promoter region of *Ugt1a1*. Western blot analysis showed that puerarin significantly inhibited AngII-induced phosphorylation of p38-MAPK. A specific inhibitor of p38-MAPK, SB203580, decreased collagen expression, and ROS generation induced by AngII in cardiac fibroblast. Together, these results suggest that puerarin prevents cardiac fibrosis via activation of Nrf2 and inactivation of p38-MAPK. Nrf2 is the key regulator of anti-fibrotic effects and upregulates metabolic enzymes UGT1A1. Autoregulatory circuits between puerarin and Nrf2-regulated UGT1A1 attenuates side effects associated with treatment, but it does not weaken puerarin’s pharmacological effects.

## Introduction

The heart manifests robust plasticity in the context of heart disease. This process is donated as pathological remodeling (Burchfield et al., 2013). Pathological myocardial remodeling is characterized by excessive accumulation of extracellular matrix, through a process called cardiac fibrosis (Kong et al., 2014; Travers et al., 2016). Physiologically, extracellular matrix provides a structural scaffold of cardiomyocytes, distributes mechanical forces throughout cardiac tissue, and mediates conduction of electrical impulses (Camelliti et al., 2005; Porter and Turner, 2009; Souders et al., 2009). Cardiac fibrosis is a final common pathway of many heart diseases. Extensive cardiac fibrosis increases myocardial stiffness, worsens diastolic function, and eventually results in progression to heart failure (Kong et al., 2014; Travers et al., 2016). Some drugs, including angiotensin-converting enzyme inhibitors, angiotensin receptor blockers, aldosterone antagonists, and β-blockers, reduce morbidity and mortality in patients with chronic systolic heart failure (Fonarow et al., 2011). However, the progression of heart failure cannot be completely suspended. New pharmacological therapies need to be discovered.

Puerarin (7,4′-dihydroxyisoflavone-8β-glucopyranoside) is a major active ingredient in the Chinese medicine *Pueraria radix* which is extracted from the kudzu root [*Pueraria lobata* (wild) Howe]. Puerarin has been widely prescribed to treat cardiovascular diseases, including hypertension (Tan et al., 2017), coronary heart disease (Xie et al., 2003) and heart failure (Duan et al., 2000). Supporting findings published previously, our laboratory has reported that puerarin may prevent cardiac fibrosis induced by pressure overload (Yuan et al., 2014; Liu et al., 2015; Tan et al., 2017). In a mouse model of cardiac fibrosis, the inhibition of myocardial fibrosis by puerarin involved transforming growth factor (TGF)-β1, monocyte chemoattractant protein (MCP)-1, and peroxisome proliferator-activated receptor (PPAR) α/γ (Chen R. et al., 2012; Tao et al., 2016). Jin et al. (2017) have demonstrated that puerarin mitigates cardiac fibrosis induced by transverse aorta constriction. This protective effect may be attributed to the upregulation of PPAR-γ and inhibition of TGF-β1/Smad2-mediated endothelial-to-mesenchymal transition. However, the effects of puerarin on cardiac fibrosis and the related mechanism remain unclear.

Puerarin is largely insoluble in water, so its oral bioavailability is low (Luo et al., 2011a,b). Understanding the metabolic pathway of puerarin may be conducive to illuminating its pharmacological effects. The results published previously by our laboratory indicated that UDP-glucuronosyltransferase (UGT) 1A1 is the primary enzyme responsible for catalysis of puerarin’s glucuronidation in human liver microsomes to form its major metabolite, puerarin-7-*O*-glucuronide (Luo et al., 2012). UGT1A1 can be upregulated by transcription factors such as transcription factor nuclear factor erythroid 2-related factor 2 (Nrf2) (Buckley and Klaassen, 2009; Gong et al., 2014). Nrf2 is a member of the cap-n-collar family of transcription factors. It is an essential modulator of celluar detoxification responses and redox status, contributing to antioxidant response element-regulated physiologic expression of numerous genes (Wang et al., 2012). Nrf2 plays an essential role in preventing fibrosis. In mice subjected to transverse aortic constriction surgery, Nrf2 deficiency exacerbated left ventricular fibrosis. Conversely, Nrf2 overexpression inhibits proliferation of cardiac fibroblasts (Li et al., 2009). In this study, we explored the roles of Nrf2 in puerarin’s preventive effect against cardiac fibrosis, as well as regulation of UGT1A1 and its pathway, in neonatal rat cardiac fibroblasts (NRCF) induced by AngII and a mouse model of cardiac fibrosis induced by abdominal aortic banding (AB).

## Materials and Methods

### Materials

Injectable puerarin was purchased from Zhejiang Zhenyuan Pharmaceutical Co., Ltd. (Shaoxing, China). Puerarin was purchased from Chengdu Must Bio-Technology Co., Ltd. (Chengdu, China). Angiotensin II was purchased from Sigma (St. Louis, MO, United States). Tert-Butylhydroquinone (tBHQ) was purchased from MedChem Express (Monmouth Junction, NJ, United StatesA). Brusatol was purchased from Tautobiotech (Shanghai, China). Cell Counting Kit-8 (CCK-8) was purchased from Dojindo Laboratories (Kumamoto, Japan). The NE-PER nuclear and cytoplasmic extraction kit, Pierce BCA Protein Assay Kit, Pierce ECL Western Blotting Substrate and Chromatin Immunoprecipitation Kit (EZ-ChIP) were purchased from Thermo Fisher (Waltham, MA, United States). RNAiso Plus, PrimeScript RT reagent Kit with gDNA Eraser (Perfect Real Time) and SYBR Premix Ex Taq II (Tli RNaseH Plus) were purchased from TaKaRa (Shiga, Japan). Reactive Oxygen Species Assay Kit was purchased from Beyotime Institute of Biotechnology (Haimen, China).

### Animal Model

Animal experiments were performed in accordance with the Guide for the Care and Use of Laboratory Animals (United States and National Institutes of Health). Specific pathogen-free Sprague-Dawley rats weighting 150–180 g (Guangdong Medical Laboratory Animal Center, Guangzhou, China) were used. The animal use and care protocol was reviewed and approved by the Ethics Committee of Guangzhou Medical University. Rats were randomly divided into three groups, each of which included six rats: the sham-operated group (Sham), abdominal aortic banding group (AB) and puerarin treatment for 6 weeks in AB rats group (Pue). Myocardial fibrosis was induced by AB (Li et al., 2014). Rats assigned to Sham group underwent a similar procedure, except for arterial ligation. Intraperitoneal injection of puerarin (50 mg/kg/day) was started from 1 week after the AB procedure. Rats in the Sham group received an equal volume of normal saline.

### Echocardiography

Following anesthetization with isoflurane, transthoracic two-dimensionally guided M-mode echocardiography was performed, 6 weeks after treatment was administered, by an experienced technician blinded to the study groups. Transthoracic echocardiography was performed with a 250 MHz ultrasound transducer (Vevo 2100, VisualSonics). Interventricular end-diastolic septum thickness (IVSd), interventricular end-systolic septum thickness (IVSs) and left ventricular posterior wall dimension (LVPWd), left ventricular internal end-diastolic diameter (LVIDd) and end-systole diameter (LVIDs) were measured. Ejection fraction (EF) and fractional shortening (FS) were then calculated as follows: EF = 100% × stroke volume/end-diastolic volume. FS = 100% × (LVIDd–LVIDs)/LVIDd.

### Organ Weight

Body weight (BW) and tibia length (TL) of each rat were measured after 6 weeks of puerarin administration. After rats were euthanized using cervical dislocation under anaesthetization, hearts were perfused briefly with 10% KCl to arrest the heart in diastole, then removed. Heart weight to body weight ratio (HW/BW) and heart weight to tibia length ratios (HW/TL) were calculated.

### Hematoxylin-Eosin (HE) Staining, Masson’s Trichrome Staining and Immunohistochemistry

After fixation with 10% formalin in phosphate-buffered saline (PBS) for 24 h, the heart tissues were subjected to alcoholic dehydration and embedded in 4% paraffin. Heart sections (5 μm) were sliced and subjected to HE and Masson’s trichrome staining. Collagen volume fraction (CVF) was determined by Image Pro Plus software to evaluate the degree of myocardial fibrosis. Mean CVF values were determined by one investigator blinded to the group assignment.

Immunohistochemical staining was performed as previously described (Li et al., 2015). Briefly, heart sections were stained with anti-collagen I antibody (1:200), anti- collagen III antibody (1:200) and anti-Nrf2 antibody (1:200) at 37°C for 2 h. After three times wash with PBS, secondary antibody was added. Then the samples were incubated at 37°C for 2 h and washed with PBS before addition of 3,3′ Diaminobenzidine (DAB) for 5 min. After hematoxylin counterstaining, dehydration in graded alcohols and stepping in xylene, neutral gum was used for mounting. Brown granules in the cells were observed by microscope (Nikon Eclipse TS 100, Japan), and six fields were chosen randomly.

### Cell Culture and Treatment

Neonatal SD rats (1–2 days old) were purchased from Guangdong Medical Laboratory Animal Center (Guangzhou, China). Primary culture of NRCFs were prepared from ventricles of neonatal rats (Yi et al., 2014). Briefly, hearts were removed from thorax and immediately placed in cold Dulbecco’s Hanks’ balanced salt solution (D-HBSS), and ventricles were minced, pooled, digested with 0.25% trypsin overnight at 4°C. At the next day, Dulbecco’s Modified Eagle Media (DMEM) medium supplemented with 10% fetal bovine serum (FBS) was added to stop the digestion. Then ventricles were digested with 1% collagenase type II and 5% bovine serum albumin (BSA) for 15 min at 37°C with rotation at the speed of 250–300 rpm. DMEM with 10% FBS were added to arrest digestion. The cells were collected and suspended in DMEM medium supplemented with 10% FBS and incubated with 95% O_2_ + 5% CO_2_. After 2 h, weakly attached or unattached cells were rinsed free and discarded, attached cardiac fibroblasts continued to culture in fresh DMEM medium supplemented with 10% FBS. When the confluence of NRCF in culture wells was up to 80–90%, the cells were digested by 0.25% trypsin and then passaged at 1:3 dilutions. And passages 2–4 were used for the subsequent experiments.

Upon reaching 50–60% confluence, the cells were treated with AngII (0.1, 1, and 10 μM) or puerarin (10, 100, and 1000 μM, dissolved in dimethyl sulfoxide [DMSO]), alone or in combination. Positive control groups were exposed to in tBHQ (50 nM, dissolved in DMSO), alone or in combination with AngII (1 μM). For inhibitor experiments, cells were treated by brusatol (100 nM, dissolved in DMSO) or SB203580 (10 μM, dissolved in DMSO).

### Transfection With Nrf2 siRNA

After plating cells in a 6-well plate or 60-mm dish, NRCF were transfected with Nrf2-siRNA or Nrf2-NC siRNA (Viewsolid Biotech, Beijing, China) for 48 h. Transfected cells were treated with AngII (1 μM) alone, AngII (1 μM)/puerarin (100 μM) or puerarin alone (100 μM) for 24 h.

### Cell Viability Analysis

Cell viability was measured by using CCK-8. Briefly, NRCF were plated in 96-well plates with a density of 5 × 10^5^cell/well. After different treatments, medium (90 μl) was incubated with 10 μl of CCK-8 solution for 2 h at 37°C in the dark. Absorbance was determined at 450 nm on a microplate reader (VARIOSKAN LUX, Thermo Scientific, United States).

### Western Blot Analysis

Whole protein extraction was prepared from NRCFs using RIPA buffer with protease and phosphatase inhibitors. The nuclear and cytoplasmic proteins were prepared using the NE-PER nuclear and cytoplasmic extraction kit (Thermo Fisher Scientific, Rockford, IL, United States), according to the manufacturer’s protocol. Bradford assay was used to measure the protein concentration (Pierce BCA Protein Assay Kit). Protein samples (20–30 μg) were separated by sodium dodecyl sulfate-polyacrylamide gel electrophoresis (SDS-PAGE) and transferred to polyvinyl difluoride (PVDF) membranes at 4°C, then blocked with 5% non-fat milk in Tris-buffered saline at room temperature for 2 h. The membranes were probed with primary antibodies including UGT1A1 (1:1000, Abcam), collagen I (1:1000, Abcam), collagen III (1:1000, Abcam), Nrf2 (1:1000, CST), p-P38 MAPK (1:1000, CST), P38 MAPK (1:1000, CST), GAPDH (1:1000, CST), β-actin (1:1000, CST), Lamin B1 (1:1000, ImmunoWay) and Keap1 (1:1000, Proteintech) overnight at 4°C with gentle shaking. After washing for three times with Tris-buffered saline containing 0.1% Tween 20 (TBST), membranes were incubated with secondary antibodies in 5% non-fat milk in TBST for 2 h at room temperature. Following three washes of the membranes, images were capture on films, which were placed in Pierce ECL Western Blotting Substrate. Further analysis was carried out using Image Pro Plus v6.0 to quantify the protein bands.

### Immunofluorescence Microscopy

Neonatal rat cardiac fibroblasts were cultured on sterile glass and treated by different agents. NRCF were washed with PBS for once and fixed with 4% formaldehyde in PBS for 15 min at room temperature. The cells were permeabilized with 0.5% Triton X-100 in PBS for 20 min and blocked with 5% goat serum for 1 h at room temperature and then incubated with anti-Nrf2 antibody (1:200, CST) overnight at 4°C. NRCF were incubated with the fluorescent secondary antibody in 3% BSA in PBS and counterstained with DAPI for 10 min at room temperature in the dark. The cells were imaged with an inverted fluorescence microscope (Nikon Eclipse Ni-u, Japan). Green fluorescence was considered a marker of Nrf2 positivity.

### Chromatin Immunoprecipitation (ChIP) Assay

Chromatin immunoprecipitation analysis was performed using a Chromatin Immunoprecipitation Kit (EZ-ChIP, Catalog ^#^ 17–371, Thermo Fisher Scientific, United States) according to manufacturer’s instruction. Briefly, cells were treated with 1% formaldehyde for 15 min for crosslinking. Then sonication was performed to shear the chromatin to a 200–1000 bp of DNA. And the size of DNA was verified by agarose gel electrophoresis. Next, chromatin samples were immunoprecipitated using anti-Nrf2 antibody (1:100, CST). Immunoprecipitated DNA was purified and amplified across the *Ugt1a1* promoter region by Real-time PCR using primers: forward: CATCCTCAAAGGGCCTGATTTAT and reverse: GGTTTCAAGATGGCAGCTGAG.

### Measurement of Intracellular Reactive Oxygen Species in Cardiac Fibroblasts

The level of intracellular reactive oxygen species (ROS) was measured using the ROSs Assay Kit. NRCF were plated in 24-well plates at a density of 5 × 10^5^cell/well. After different treatments, medium was removed, and the cells were washed with PBS. A solution of 10 μM fluorescent probe 2′,7′-dichlorofluorescin diacetate (DCFH-DA) in protein-, serum-free medium was added for 30 min at 37°C in the dark. Then intracellular ROS were detected by immunofluorescence microscope. The OD value of intracellular ROS was also checked by fluorometer in opaque-walled 96 well plates after different treatment.

### Statistical Analyses

Data were expressed as the means ± standard error (SEM). The differences in means between groups were evaluated using one-way analysis of variance (ANOVA), followed by the Tukey-Kramer HSD *post hoc* test for multiple comparisons. Differences with *p* < 0.05 were considered statistically significant.

## Results

### Puerarin Inhibited AB Induced-Cardiac Fibrosis in Rats

**FIGURE 1:**
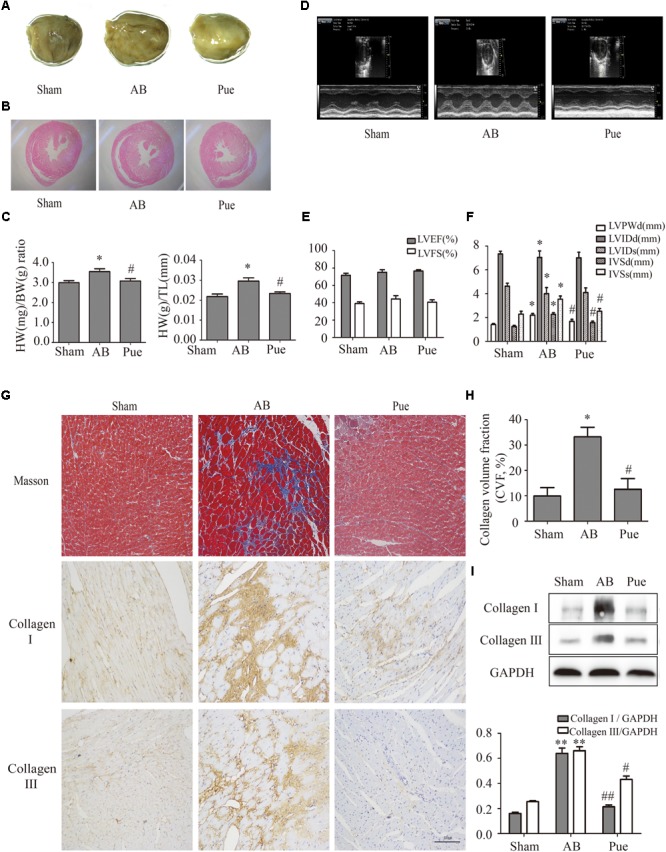
Puerarin protected against abdominal aortic banding (AB)-induced cardiac fibrosis. **(A)** Gross hearts. **(B)** HE staining. **(C)** Heart weight/body weight (HW/BW) ratio and heart weight/tibial length (HW/TL) ratio. **(D)** Representative echocardiographic images. **(E)** Left ventricular function. **(F)** Indexes of cardiac structure. **(G)** Masson trichrome staining and immunohistochemical staining of collagen I and III. **(H)** Collagen volume fraction (CVF) of Masson trichrome staining. **(I)** Quantitative analysis of collagen I and collagen III *in vivo*. Sham, sham-operated group; AB, aortic banding group; Pue, puerarin-treated aortic banding group. ^∗^*P* < 0.05 vs. Sham, ^∗∗^*P* < 0.01 vs. Sham, ^#^*P* < 0.05 vs. AB, ^##^*P* < 0.01 vs. AB. *n* = 6 for each group.

Rats subjected to AB surgery 7 weeks showed cardiac hypertrophy and myocardial remodeling as evidenced by increased cardiac mass (**Figure 1A**), myocyte cross sectional area (**Figure 1B**), heart weight/body weight (HW/BW) ratio, and heart weight/tibial length (HW/TL) ratio (**Figure 1C**) compared to sham. These measurements were significantly decreased in puerarin-treated rats. Comparison of ultrasonic data (**Figures 1D,E,F**) between groups revealed no obvious trend in left ventricular ejection fraction (LVEF) or left ventricular fractional shortening (LVFS) (**Figure 1E**). Compared to Sham, AB animals showed increased left ventricular posterior wall dimension (LVPWd), interventricular end-diastolic septum thickness (IVSd), and interventricular end-systolic septum thickness (IVSs). However, AB animals showed decreased left ventricular internal end-diastolic diameter (LVIDd) and end-systole diameter (LVIDs). Puerarin could reverse these changes in LVPWd, IVSd, and IVSs, but not in LVIDd and LVIDs (**Figure 1F**). AB rats also exhibited manifest cardiac fibrosis as evidenced by collagen deposit, increase of collagen volume fraction (**Figures 1G,H**), and increasing collagen I and collagen III (**Figures 1G,I**). Puerarin significantly attenuated cardiac fibrosis response induced by AB (**Figures 1G–I**).

### Puerarin Inhibited the Proliferation of Cultured Neonatal Rat Cardiac Fibroblasts

**FIGURE 2:**
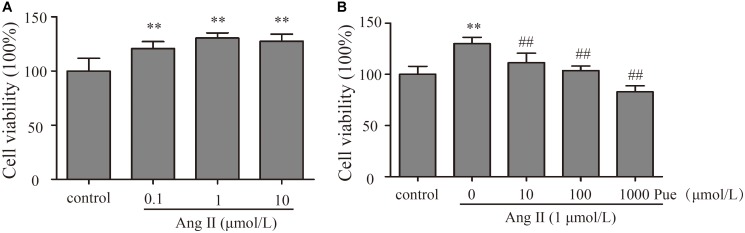
Puerarin decreased proliferation of cultured NRCF. The CCK-8 assay was used to detect proliferation of NRCF. **(A)** Effects of various concentrations of AngII (0.1–10 μM) on proliferation of NRCF. **(B)** Effects of various concentrations of puerarin (10–1000 μM) on AngII-induced proliferation of NRCF. ^∗∗^*P* < 0.01 vs. control, ^#^*P* < 0.05 vs. AngII, ^##^*P* < 0.01 vs. AngII. *n* = 8 for each group.

In order to investigate the mechanism of puerarin protecting against cardiac fibrosis, we did some experiments in cardiac fibroblasts. First of all, we explored the effective concentration of AngII and puerarin by CCK-8 assay. NRCF were treated with different concentrations of AngII (0.1–10 μM) for 24 h. The results showed that 1 μM AngII significantly promoted the cell proliferation (**Figure 2A**) as similar to the previous report (Stacy et al., 2007). So, 1 μM of AngII was selected to establish a cell model of cardiac fibrosis. Then, NRCF were pre-incubated with various concentrations of puerarin (10–1000 μM) for 24 h. 1000 μM of puerarin reduced the cell viability, but not for 1–100 μM of puerarin (Supplementary Figure S2). Treatment with puerarin inhibited AngII-induced cell proliferation of NRCF. This effect was concentration-dependent (**Figure 2B**). Based on the results, a 100 μM dose of puerarin was used for subsequent experiments. Similar concentration was selected in other *in vitro* studies (Yeung et al., 2006; Chen Y.-Y. et al., 2012).

### Puerarin Protected Against Cardiac Fibrosis Through Nrf2/ROS Pathway in Cultured Neonatal Rat Cardiac Fibroblasts

**FIGURE 3:**
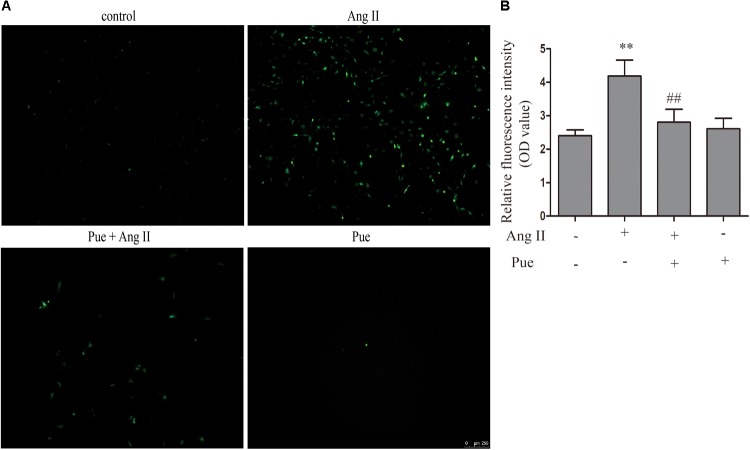
Puerarin decreased intracellular ROS induced by AngII. **(A)** Intracellular ROS were observed by fluorescence microscopy. **(B)** Quantitative analysis of intracellular ROS in NRCF by fluorometry. AngII, 1 μM; puerarin, 100 μM. ^∗∗^*P* < 0.01 vs. control, ^##^*P* < 0.01 vs. AngII. *n* = 6 for each group.

It is widely agreed that oxidant stress participated in the activation or differentiation of fibroblasts. We used AngII to induce oxidative stress, and ROS was detected by fluorescent probe DCFH-DA and the relative fluorescence intensity (OD value) was assessed. As shown in **Figure 3**, the relative fluorescence intensity of ROS increased significantly when NRCF were treated with AngII. Puerarin, pretreating before AngII administration, decreased the relative fluorescence intensity of ROS in NRCF.

**FIGURE 4:**
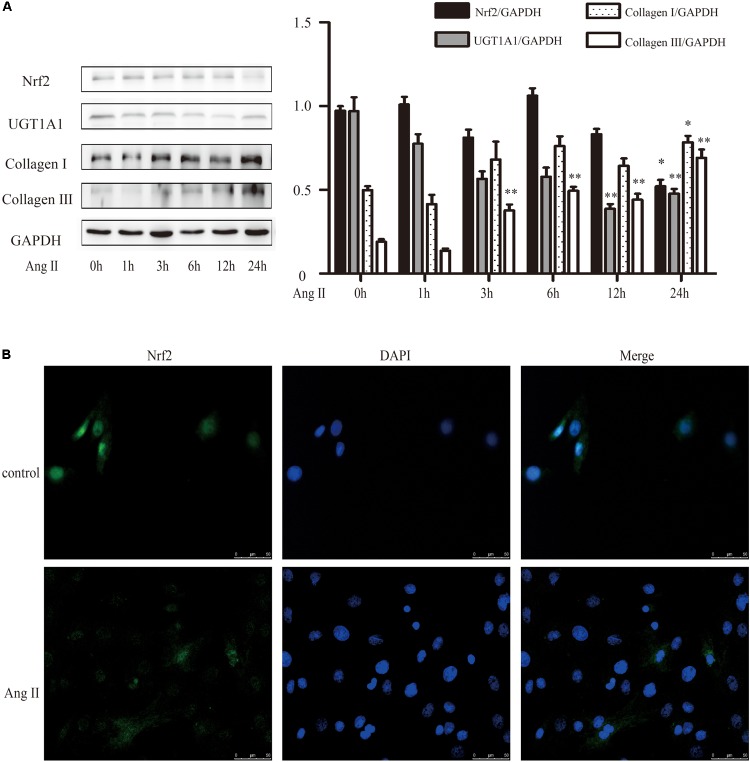
AngII promoted cardiac fibrosis in cultured neonatal rat cardiac fibroblasts. **(A)** Representative western blots and quantitative analysis of Nrf2, UGT1A1, collagen I and collagen III; **(B)** immunofluorescence analysis of AngII on Nrf2. AngII, 1 μM. ^∗^*P* < 0.05 vs. 0 h, ^∗∗^*P* < 0.01 vs. 0 h.

Nrf2 mediates the cell’s reaction to oxidative stress by binding to an antioxidant responsive element (ARE). Loss of Nrf2 results in increased susceptibility to reactive oxygen in both cardiac fibroblasts and cardiomyocytes (Li et al., 2009). A detailed examination of the time courses of the effect of AngII on Nrf2, collagen I and collagen III were done. It revealed a time-dependent downregulation of protein level of Nrf2 after exposure to AngII (**Figure 4A**). And conversely, a notable raise of collagen I and collagen III expression after AngII treatment (**Figure 4A**). The immunofluorescence results also showed that AngII reduced the expression of Nrf2 in the nucleus after exposure to AngII for 24 h (**Figure 4B**).

**FIGURE 5:**
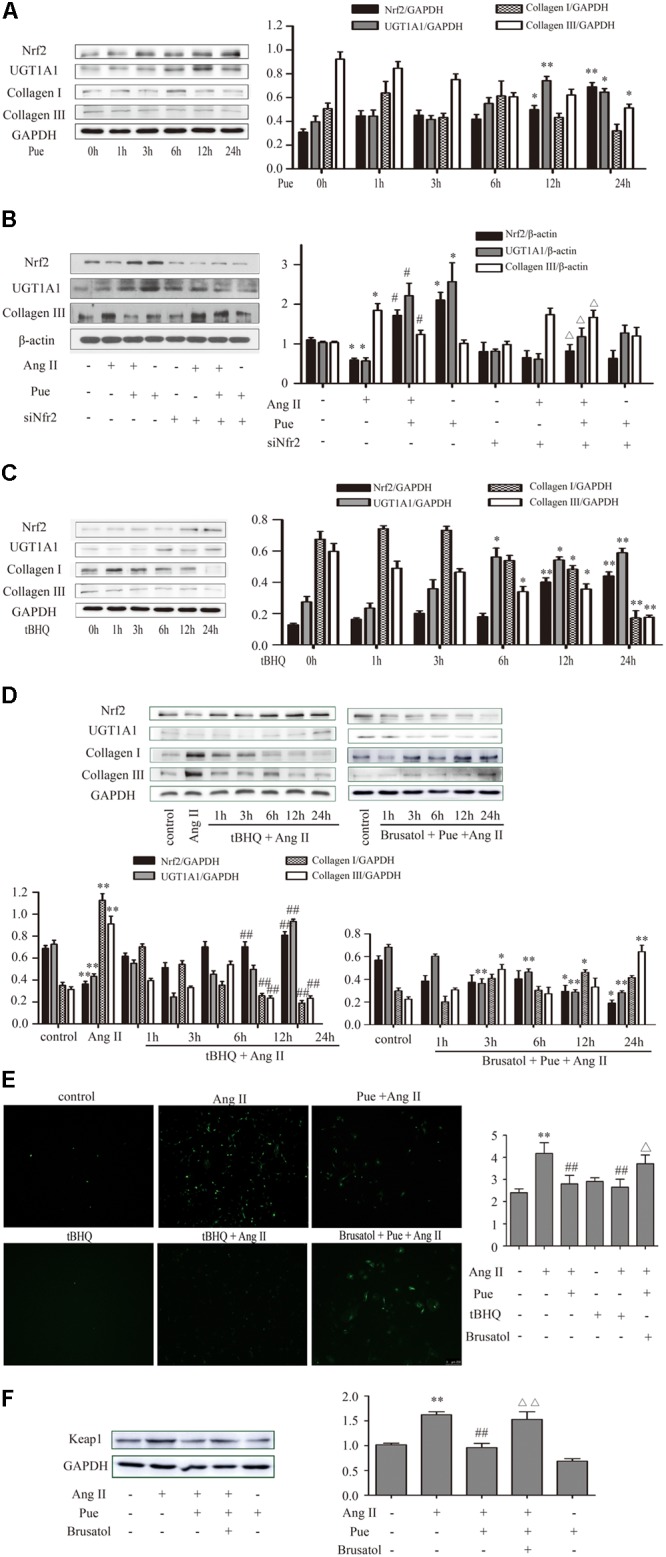
Puerarin protected against AngII induced-cardiac fibrosis and oxidative stress in cultured neonatal rat cardiac fibroblasts. **(A–D)** Representative western blots and quantitative analysis of Nrf2, UGT1A1, collagen I, and collagen III. **(A)** The time courses of puerarin effects on cultured neonatal rat cardiac fibroblasts. **(B)** The time courses of effects of tBHQ (an agonist of Nrf2) on cardiac fibrosis. **(C)** Puerarin protected against AngII induced-cardiac fibrosis. **(D)** The time courses of effects of tBHQ and Brusatol (an inhibitor of Nrf2) on AngII induced-cardiac fibrosis. **(E)** Puerarin protected against AngII induced- intracellular ROS (by fluorescence microscopy and fluorometer). **(F)** Puerarin inhibited the expression of Keap1 in cultured neonatal rat cardiac fibroblasts. Representative western blots and quantitative analysis of Keap 1. AngII, 1 μM; puerarin, 100 μM; tBHQ, 50 nM; Brusatol, 100 nM; siNrf2, Nrf2 specific siRNA. ^∗^*P* < 0.05 vs. control (0 h), ^∗∗^*P* < 0.01 vs. control (0 h), ^#^*P* < 0.05 vs. AngII, ^##^*P* < 0.01 vs. AngII, ▵ *P* < 0.05 vs. Pue + AngII, ▵ ▵ *P* < 0.01 vs. Pue + AngII. *n* = 6 for each group.

We then determined the role of puerarin on Nrf2 expression and cardiac fibrosis. We found that compared with AB group, puerarin promoted the expression of Nrf2 in heart tissue (Supplementary Figure S1). As shown in **Figure 5A**, the time courses of the effect of puerarin on cardiac fibroblasts indicated an increased expression of Nrf2 in time-dependent manner, and a decreased expression of collagen III. After co-incubation of puerarin and AngII 24 h, puerarin markedly increased the protein level of Nrf2 in AngII-treated NRCF, while it decreased the protein level of collagen III (**Figure 5B**). Similar to puerarin, an agonist of Nrf2, tBHQ alone or co-treatment with AngII promoted higher protein expression of Nrf2 in a time-dependent manner in NRCF while notably downregulated collagen I and collagen III (**Figures 5C,D**). These data indicated that puerarin probably activated Nrf2 to attenuate AngII-induced cardiac fibrosis.

To confirm that whether Nrf2 participated in the protective effect of puerarin against cardiac fibrosis, cardiac fibroblasts were exposed to Nrf2 siRNA or AngII, Brusatol (an inhibitor of Nrf2) and AngII alone or combination with puerarin. Results shown a markedly depression of Nrf2 and significant raise of collagen I and collagen III in 24 h after exposure to coincubation of siNrf2, puerarin and AngII (**Figure 5B**), or co-treatment of brusatol, puerarin and AngII (**Figure 5D**) in NRCF.

We further detected whether Nrf2 participated in the antioxidant effects of puerarin. As shown in **Figure 5E**, AngII treatment increased the relative intensity of ROS, which was blocked by puerarin treatment. tBHQ also could decreased the relative fluorescence intensity of ROS induced by AngII. Brusatol blocked the effects of puerarin.

**FIGURE 6:**
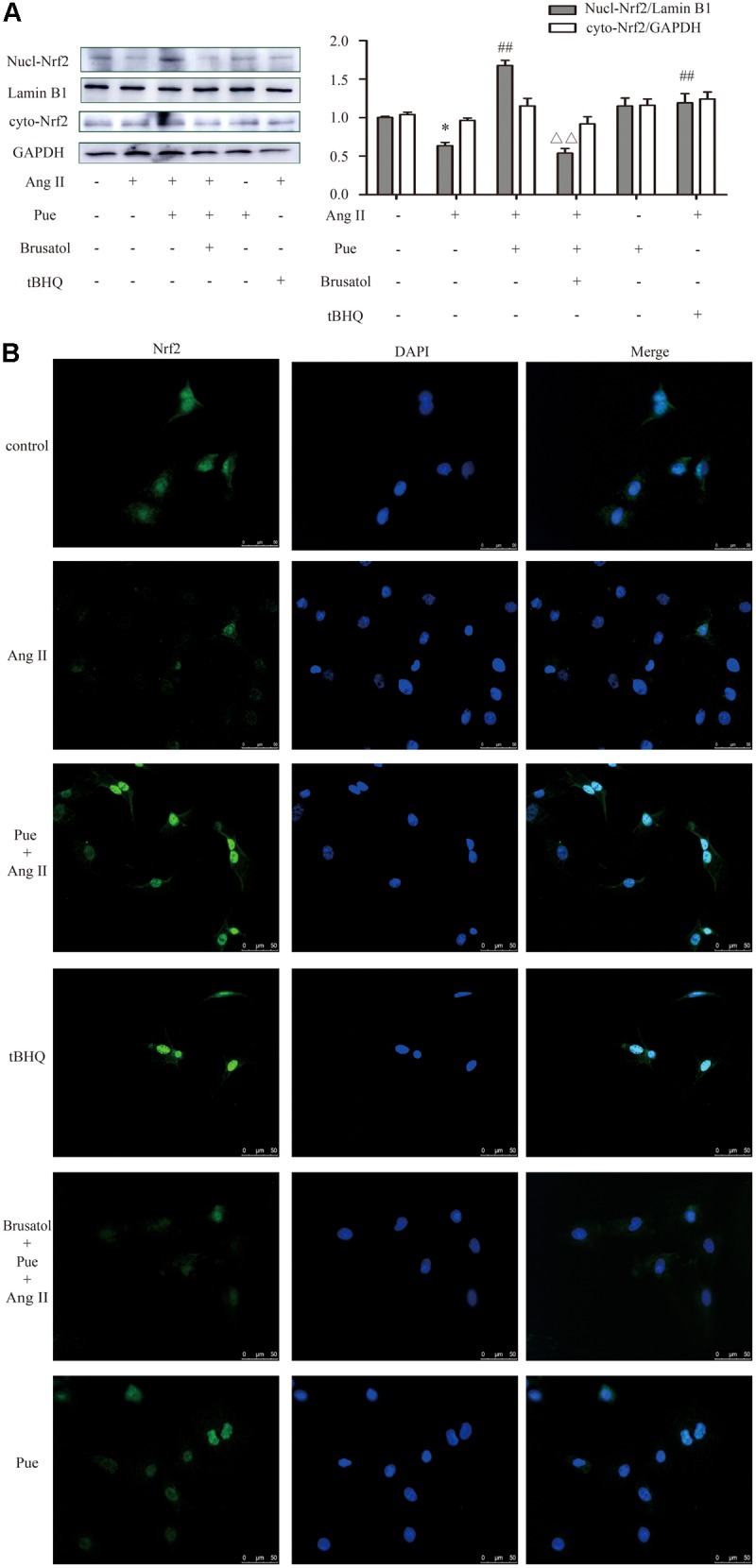
Puerarin promoted the expression of Nrf2 in cultured neonatal rat cardiac fibroblasts. **(A)** Representative western blots and quantitative analysis of Nrf2 in nucleus and cytoplasm. **(B)** Representative fluorescence images showed the expression of Nrf2 in nucleus. AngII, 1 μM; puerarin, 100 μM; tBHQ, 50 nM; Brusatol, 100 nM. ^∗^*P* < 0.05 vs. control, ^##^*P* < 0.01 vs. AngII, ▵ ▵ *P* < 0.01 vs. puerarin + AngII. *n* = 6 for each group.

Under normal conditions, Nrf2 is bound in the cytoplasm to Kelch-Like ECH-Associated Protein 1 (Keap 1). Upon stimulation, Nrf2 escapes from Keap 1-mediated repression and is translocated to the nucleus (Gao et al., 2015). Ang II increased the protein level of Keap 1 in NRCF, which was reversed by puerarin. Brusatol blocked the effect of puerarin (**Figure 5F**). The western blot of nuclear protein and immunofluorescence results also revealed that both puerarin and tBHQ provoked the expression of Nrf2 in the nucleus, and brusatol reversed the increase of Nrf2 in the nucleus induced by puerarin, compared with AngII group. But there was no obvious change in the expression of Nrf2 in the cytoplasm between different groups (**Figure 6** and Supplementary Figure S4). It indicated that puerarin enhanced the expression in NRCF treated with Ang II. Simultaneously, puerarin promoted the activation of Nrf2 via downregulation of Keap 1 and translocation of Nrf2 to nucleus. These results suggest that Nrf2/ROS pathway may be an important route for puerarin to fight against cardiac fibrosis.

### Puerarin Upregulated UGT1A1 Levels Through Activation of Nrf2 in Cultured Neonatal Rat Cardiac Fibroblasts

UGT1A1 is one of UDP-glucuronosyltransferases. Our previous study has reported that UGT1A1 significantly catalyzed the formation of puerarin metabolites, and its activity was significantly higher than other catalyzing enzyme (Luo et al., 2012). We detected UGT1A1 expression by Western blotting in NRCF which were subjected different treatments. The protein level of UGT1A1 were dramatically upregulated by puerarin or tBHQ treatment in a time-dependent manner (**Figures 5A,C**). In contrast, a significant decrease in UGT1A1 was observed in NRCF from 3 to 24 h after exposure to AngII (especially at 12 and 24 h) (**Figure 4A**). UGT1A1 upregulation was clearly observed after 24 h co-incubation with puerarin and AngII. After siRNA or inhibitor specific downregulation of Nrf2, the puerarin-induced upregulations of UGT1A1 was partially abolished (**Figures 5B,D**). These data suggested that puerarin upregulated the expression of UGT1A1 via transcription factor Nrf2.

**FIGURE 7:**
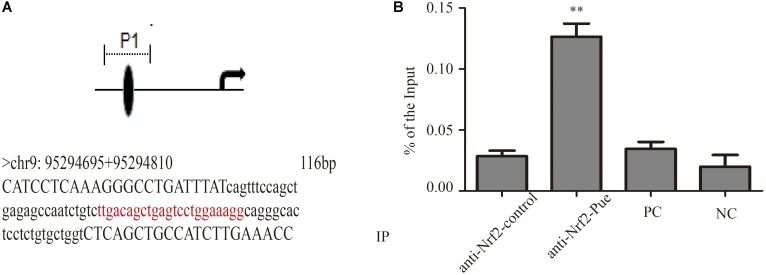
Puerarin promoted Nrf2 expression and blinded to *Ugt1a1* promoter regions. **(A)**: The direct binding sites in *Ugt1a1* promoter (red marker). **(B)** *In Vivo* characterization of Nrf2 binding to the *Ugt1a1* promoter by ChIP assay. Chromatin from cardiac fibroblasts was immunoprecipitated with Nrf2 antibody (anti-Nrf2 control and anti-Nrf2-Pue) or without antibody (positive control, PC; negative control, NC), then amplified by q-PCR. ^∗∗^*P* < 0.01 vs. anti-Nrf2 control *n* = 3 for each group.

In order to confirm that puerarin increased expression of UGT1A1 via Nrf2, we studied contribution of Nrf2 to *Ugt1a1*, gene expression using ChIP. The ChIP results shown that puerarin significantly increased Nrf2- associated *Ugt1a1* promoter activity (**Figure 7**, Supplementary Figure S5, and Supplementary Table S1).

### Puerarin Protected Against Cardiac Fibrosis Through p38 MAPK in Cultured Neonatal Cardiac Fibroblasts

**FIGURE 8:**
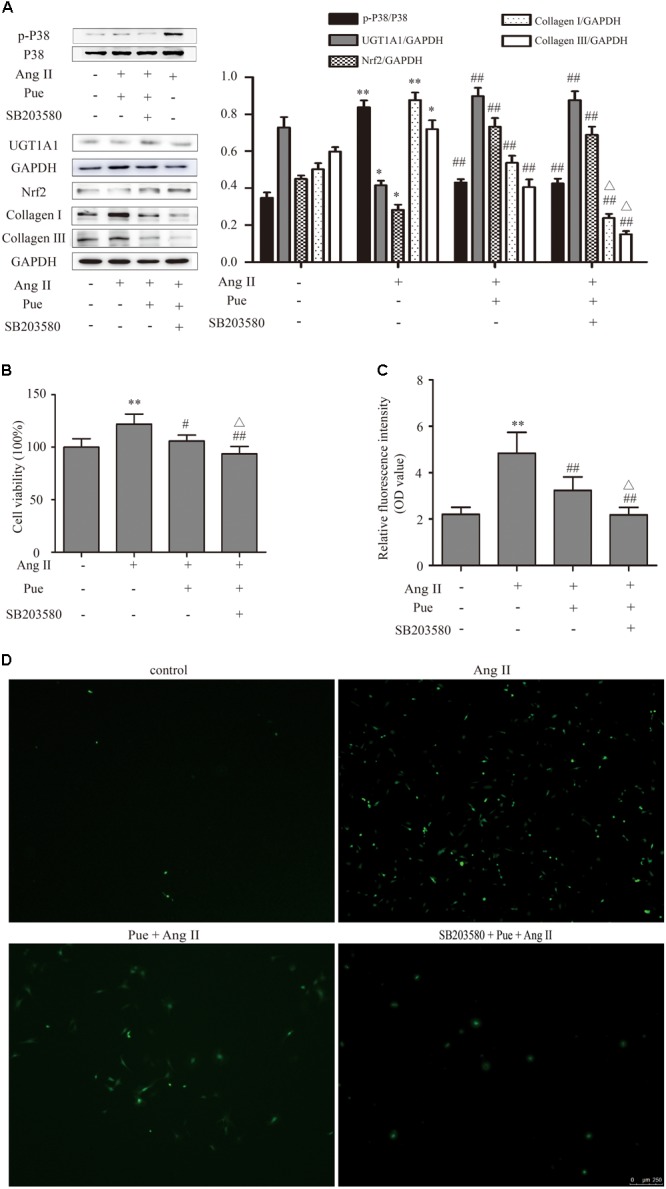
p38-MAPK signal participated in regulating puerarin on cardiac fibrosis *in vitro*. **(A)** Representative western blots and Quantitative analysis of p38-MAPK, Nrf2, UGT1A1, collagen I, and collagen III in puerarin protecting against AngII-induced cardiac fibrosis. **(B)** Effects of SB203580 (inhibitor of p38-MAPK) on proliferation of NRCF by CCK-8 assay. **(C,D)** SB203580 inhibited AngII-induced cardiac fibrosis in intracellular ROS (by fluorometer and fluorescence microscopy). AngII, 1 μM; puerarin, 100 μM; SB203580, 10 μM. ^∗^*P* < 0.05 vs. control (0 h), ^∗∗^*P* < 0.01 vs. control (0 hr), ^#^*P* < 0.05 vs. AngII, ^##^*P* < 0.01 vs. AngII, △*P* < 0.05 vs. Pue + AngII. *n* = 6 for each group.

p38-MAPK exerts an important impact on the proliferation of NRCF. We investigated whether p38-MAPK participated in anti-fibrotic effect of puerarin. The results shown that AngII significantly increased phosphorylation of p38-MAPK in NRCF, and puerarin significantly decreased the protein level of phosphorylated p38-MAPK (**Figure 8A**). The protein level of p38-MAPK did not show difference in each group (**Figure 8A**). SB203580, a specific inhibitor of p38-MAPK, decreased protein levels of collagen I and collagen III, inhibit the proliferation of NRCF induced by AngII basing on puerarin administration (**Figures 8A,B**). Similar effect of SB203580 on ROS generation was also observed (**Figures 8C,D**). These results indicated that puerarin prevented the proliferation and oxidative stress of NRCF induced by AngII at least partly via inactivation of p38-MAPK. Some studies reported that p38-MAPK was involved in regulation of the phosphorylation and activation of Nrf2 (Ho et al., 2011). In our study, in the present of puerarin, levels of phosphorylation of p38-MAPK decreased. The upregulation of Nrf2 and UGT1A1 in puerarin treated-NRCF persisted after 24-h exposure to SB203580 24 h. Thus, in the context of cardiac fibrosis, the activation of Nrf2 induced by puerarin is likely independent of the p38-MAPK pathway.

## Discussion

Cardiac fibrosis is an important component of cardiac remodeling that may be related to adverse cardiovascular outcomes (Chin et al., 2016). A previous study from our laboratory reported that puerarin decreased deposition of collagen in hypertrophy rats (Liu et al., 2015). In the present study, experiments in rats with AB-induced cardiac fibrosis indicated that puerarin could significantly reduce the deposition of collagen I and collagen III.

To investigate the mechanisms underlying puerarin’s protective effect against cardiac fibrosis, NRCF treated with AngII were used as an *in vitro* model of cardiac fibrosis (Fujita et al., 2008). We found that puerarin significantly prevented the proliferation of NRCF induced by AngII, and reduced AngII-induced increases in expression of collagen I and collagen III. AngII may induce ROS generation and oxidative stress (Lijnen et al., 2012), and the latter of which is involved in cardiac fibrosis (Wu et al., 2016). We have reported that puerarin may prevent oxidative stress in neonatal rat cardiomyocytes induced by AngII (Hou et al., 2017). In the present study, puerarin also decreased ROS generation in NRCF induced by AngII.

Nrf2 is a member of the cap-n-collar family of transcription factors. Under physiologic conditions, Nrf2 is retained in the cytoplasm. Upon activation, Nrf2 rapidly translocates to the nucleus, where it binds to the antioxidant response element (ARE) in the upstream promoter region. Binding promotes transcription of a battery of antioxidant genes (Kensler et al., 2007; Zhou et al., 2014). Accumulating data indicate that cardiac fibrosis involves signaling pathway mediated by Nrf2 (Li et al., 2009, 2016). AngII decreased protein expression of Nrf2 in NRCF. In NRCF treated with AngII, puerarin significantly increased protein expression of Nrf2, accompanied by decreases in levels of collagen I, III, and ROS. Immunofluorescence and western blot analyses showed that puerarin increased nuclear levels of Nrf2. Brusatol, an inhibitor of Nrf2, reversed these effects. These results indicated that puerarin enhanced the expression of Nrf2, as well as tansclocation of Nrf2 into nucleus. These results suggest that puerarin prevented cardiac fibrosis via Nrf2/ROS pathway. Interestingly, protein levels of UGT1A1, a major metabolic enzyme of puerarin, exhibited a pattern similar to that of Nrf2 protein expression in NRCF treated by AngII, AngII + puerarin, AngII + puerarin + brusatol. The results of ChIP assay confirmed that puerarin enhanced Nrf2 binding to the *Ugt1a1* promoter in NRCF. In rats, puerarin promotes the expression of UGT1A1 via activation of Nrf2. Nrf2 was the common transcription factor for puerarin to protect against cardiac fibrosis and upregulate the metabolic enzyme UGT1A1.

After administration of puerarin, drug levels must remain relatively constant. Severe adverse events may occur after intravenous injection of puerarin (Hou et al., 2011). In the present study, we found that puerarin upregulated its major metabolic enzyme UGT1A1 via activation of Nrf2. This autoregulatory circuit helps to maintain the concentration of puerarin within appropriate limits. Certainly, upregulation of UGT1A1 catalyzes metabolism of puerarin to puerarin-7-*O*-glucuronide. The anti-hypertrophy effect of puerarin-7-*O*-glucuronide is similar to that of its precursor, puerarin. Therefore, the autoregulatory circuit between puerarin and Nrf2-regulated UGT1A1 do not weaken its pharmacological effects.

The p38-MAPK pathway strongly affects proliferation of NRCF and may be activated by oxidative stress (Akiyama-Uchida et al., 2002; Yin et al., 2015; Hu et al., 2016). In our study, puerarin downregulated phosphorylation of p38-MAPK induced by AngII, but not p-ERK1/2 or p-JNK (Supplementary Figure S3). In the presence of a p38-MAPK inhibitor, protein levels of phosphorylated p38-MAPK in AngII-treated NRCF could not be further inhibited by puerarin. Conversely, Nrf2 protein expression could not be further increased by puerarin. These findings suggested that puerarin prevented cardiac fibrosis induced by Ang II at least partly via inactivation of p38-MAPK, and the activation of Nrf2 by puerarin is likely independent of the p38-MAPK pathway in NRCF. The decreased p38-MAPK phosphorylation (induced by AngII) inhibited collagen expression and cell proliferation in NRCF.

It has been demonstrated that the activation of Nrf2 typically involves the ERK and p38-MAPK pathways. Chen L. et al. (2012) reported that curcumin induced activation of Nrf2 in a p38-dependent manner. Ho et al. (2012) also observed that diallyl sulfide activated Nrf2-driven ARE activation via the p38 pathway. In contrast, some investigators also found that p38-MAPK plays a negative role on Nrf2 activation. It has been reported that activated Nrf2 may induce expression of antioxidant-genes and inhibit expression of adhesion molecule by decreasing phosphorylation/activation of p38 (Huang et al., 2001). Ma et al. (2014) shown that puerarin induced activation of Nrf2 and inhibited phosphorylation of ERK in carbon tetrachloride-induced cell death in mouse kidney. Our study also shown that puerarin activated Nrf2 and inhibit phosphorylation of p38 in AngII-treated NRCF. Hence, it is important to discriminate crosstalk among various signaling pathways involved in the cardioprotective effects of puerarin.

## Conclusion

**FIGURE 9:**
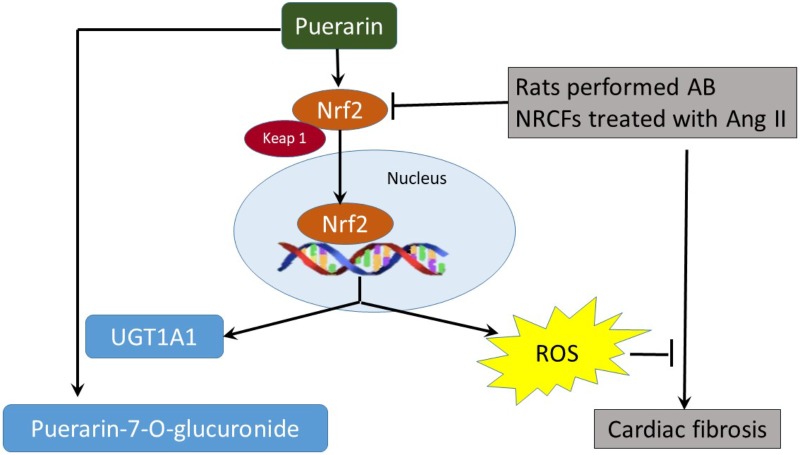
The proposed autoregulatory circuit between puerarin and Nrf2-regulated UGT1A1.

Puerarin prevents cardiac fibrosis via downregulation of Keap 1, promoting expression of Nrf2 and its nuclear translocation. The inactivation of p38-MAPK also contributes to the anti-fibrotic effects of puerarin. Nrf2 is the key regulator of anti-fibrotic effects and upregulates metabolic enzymes UGT1A1 in NRCF (**Figure 9**).

## Author Contributions

M-SC and C-FL conceived and designed the experiments. S-AC and G-JZ performed the experiments and analyzed the data. X-WL, Y-YH, H-LL, Y-QH, L-RL, YH, and C-WO contributed reagents, materials, and analysis tools. C-FL and NH wrote the paper.

## Conflict of Interest Statement

The authors declare that the research was conducted in the absence of any commercial or financial relationships that could be construed as a potential conflict of interest.
