# Supplementary material for: Nrf2 Is a Key Regulator on Puerarin Preventing Cardiac Fibrosis and Upregulating Metabolic Enzymes UGT1A1 in Rats
Source: Front Pharmacol. 2018 Jun 6;9:540. doi: 10.3389/fphar.2018.00540 (PMC5997811; doi:10.3389/fphar.2018.00540)
Supplement: Supplementary file 1 [file Presentation_1.pdf]

Supplement

Supplement 1

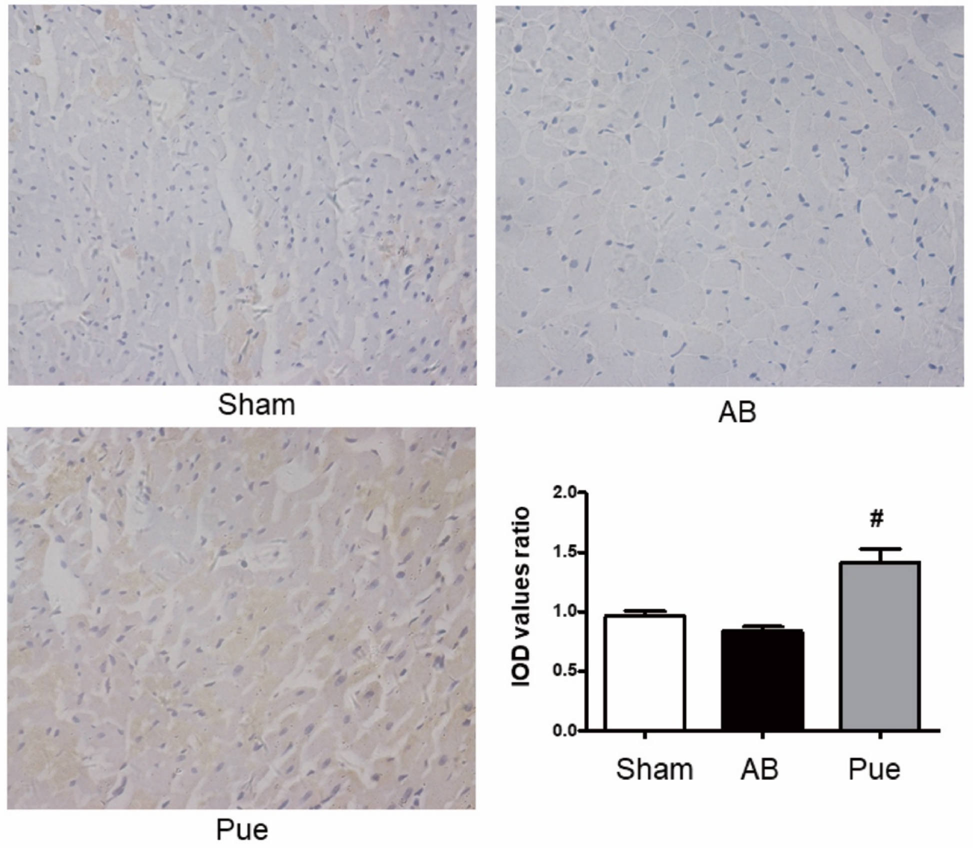

Fig S1 Immunohistochemical staining for Nrf2 in sections of animal tissue. $^{\#}P < 0.05$ *vs.* AB group.

Supplement 2

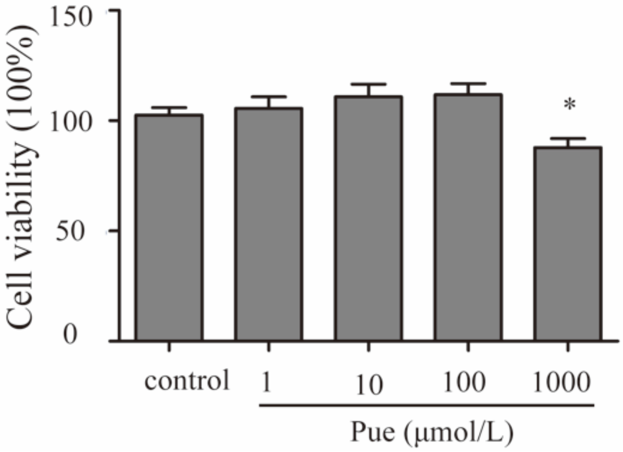

Fig. S2 The CCK-8 assay was used to detect proliferation of neonatal rat cardiac fibroblasts (NRCF), so as to evaluate the toxicity of puerarin. *P < 0.05 *vs.* control, Pue 1 μmol/L, Pue 10 μmol/L, and Pue 100 μmol/L.

Supplement 3

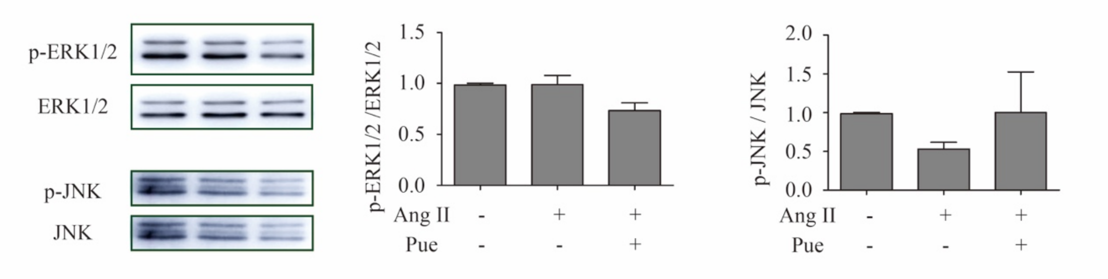

Fig. S3 Neither ERK1/2 nor JNK regulates the effects of puerarin in protecting against cardiac fibrosis in vitro. Ang II, 1 μM; puerarin, 100 μM. n = 6 for each group.

Supplement 4

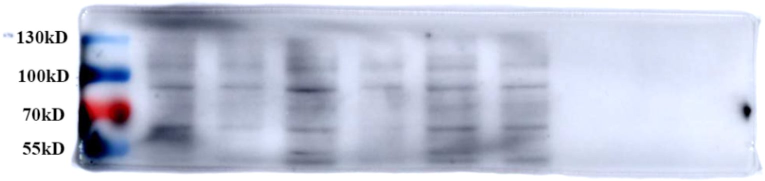

Fig. S4 The primitive strip of nuclear Nrf2 corresponding to Figure 6A.

Supplement 5

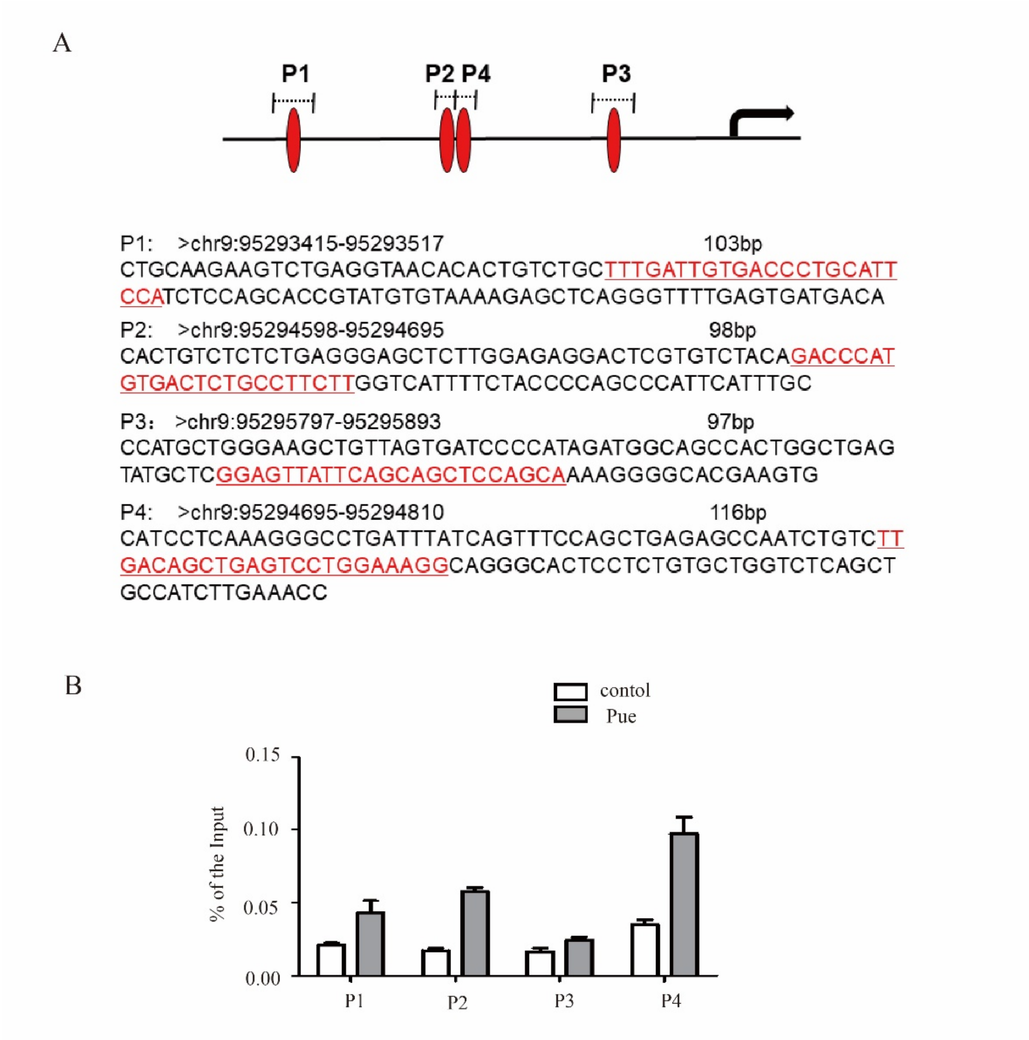

Fig. S5 The combination of transcription factor Nrf2 with the target gene promoter region P1 region, P2 region, P3 region, and P4 region in the control group and puerarin treatment group, respectively. A: Different binding sites in *Ugt1a1* promoter. B: In Vivo characterization of Nrf2 binding to the *Ugt1a1* promoter by ChIP assay.

Table S1

Region	Primer of *Ugt1a1*
P1	*Ugt1a1*-F: 5′-CTGCAAGAAGTCTGAGGTAACACAC-3′
	Ugt1a1-R: 5′-TGTCATCACTCAAAACCCTGAGC-3′
P2	*Ugt1a1*-F: 5′-CACTGTCTCTCTGAGGGAGCTCTTG-3′
	Ugt1a1-R: 5′-GCAAATGAATGGGCTGGGGTAG-3′
P3	*Ugt1a1*-F: 5′-CCATGCTGGGAAGCTGTTAGTGATC-3′
	Ugt1a1-R: 5′-CACTTCGTGCCCCTTTTGCTG-3′
P4	*Ugt1a1*-F: 5′-CATCCTCAAAGGGCCTGATTTAT-3′
	Ugt1a1-R: 5′-GGTTTCAAGATGGCAGCTGAG-3′

Table S1 the primer of *Ugt1a1* for different region
